# Acute multiple brain infarctions associated with Streptococcus suis infection: a case report

**DOI:** 10.1186/s12879-024-09318-9

**Published:** 2024-04-26

**Authors:** Wenxin Wei, Zhenhu Qiao, Donghua Qin, Yu Lan

**Affiliations:** Department of Neurology, Minzu Hospital Affiliated of Guangxi Medical University, Nanning, Guangxi 530001 China

**Keywords:** Streptococcus suis, Cerebral infarction, Cognitive impairment, Meningitis, Sepsis

## Abstract

*Streptococcus suis* is one of the most common zoonotic pathogens, in humans and can cause meningitis, endocarditis, arthritis and sepsis. Human cases of *Streptococcus suis* infection have been reported worldwide, and most of those cases occurred in Asia. Hearing loss is the most common sequela of *Streptococcus suis* meningitis. *Streptococcus suis* infection complicated with acute cerebral infarction has rarely been reported. Therefore, to provide a reference for this disease, we reported a case of acute multiple brain infarctions associated with *Streptococcus suis* infection. In our report, a 69yearold male patient had *Streptococcus suis* meningitis and sepsis, which were associated with multiple acute cerebral infarctions in the pons and bilateral frontotemporal parietal occipital lobes. After treatment, the patient exhibited cognitive impairment, dyspraxia and irritability. There are limited case reports of cerebral infarction associated with *Streptococcus suis* infection, and further research is needed to determine the best treatment method.

## Introduction

*Streptococcus suis* is one of the most common zoonotic pathogens in humans and can cause meningitis, endocarditis, arthritis and sepsis after direct contact with pigs or pork [1–3]. Patients with severe infection may present with Streptococcal Toxic Shock-Like Syndrome (STSLS) or Streptococcus meningitis syndrome. Human cases of *S. suis* infection have been reported worldwide, and most of these cases are in Asia [3]. *S. suis* strains are classified serologically into 35 serotypes; serotype 2 is the most virulent and is closely associated with human and pig diseases [3]. Hearing loss is the most common sequela of *S. suis* meningitis [2, 3]. Patients may also present with other neurological sequelae, such as cognitive impairment, tinnitus and other symptoms [2]. This article reports a case of *Streptococcus suis* infection associated with acute cerebral infarction.

## Case presentation

A 69-year-old male patient was admitted to the hospital on 22 September 2022 due to fever lasting for 4 h and coma for 2.5 h. At midnight on 22nd September the patient developed vomiting and loss of consciousness. Then, the spouse called 120 and took him to the emergency department of our hospital. The patient was soon admitted to our Department of Neurology due to an unexplained coma. The patient was a retired corporate executive, and his job was not associated with pigs or pork. The patient cut his left hand with a kitchen knife before onset, and this could have led to an infection. The spouse denied that the patient had any history of hypertension, heart disease, or diabetes. The spouse denied that the patient had a history of substance abuse. Physical examination after admission revealed a body temperature of 39.5℃, pulse rate of 142 beats/minute, respiratory rate of 45 breaths/minute, and blood pressure of 188/102 mmHg. His Glasgow coma scale score was 6 points. There was a 0.5 cm wound on the left index finger. His pupils were equal in size, and his pupillary reflexes were intact. There was no signs of meningeal irritation. The muscle strength of the right upper limb was roughly measured at grade 3. The right lower limb and left limb could not be lifted and could only move on the bed, so the muscle strength of the right lower limb and left limb were roughly measured at grade 2. The extremity tendon reflexes of the patient were normal, and he had a negative bilateral Babinski sign. His National Institutes of Health Stroke Scale (NIHSS) score was 24 points. Because the patient had shortness of breath, the patient was transferred to the intensive care unit (ICU) a few hours after admission. He was mechanically ventilated and underwent lumbar puncture.

His routine blood test results were as follows: white blood cell count, 16.27 × 10^9^/L; neutrophil ratio, 90.8%; neutrophil count, 14.77 × 10^9^/L; hemoglobin, 141 g/L; and platelet count, 128 × 10^9^/L. The creatinine concentration was 107 µmol/L, the serum procalcitonin concentration was 32 ng/mL, the lactic acid concentration was 10.2mmol/L, the glucose concentration was 21.3 mmol/L, the cholesterol concentration was 5.24 mmol/L, the triglyceride concentration was 2.59 mmol/L and the low-density lipoprotein was 2.82 mmol/L. The patient had normal liver function. A cerebrospinal fluid analysis revealed a glucose level of 1.4 mmol/L, a protein concentration of 1.46 g/L, a chloride concentration of 101 mmol/L, a white blood cell count of 4400 × 10^6^/L and a cerebrospinal fluid pressure of 330 mmH_2_O. Blood and cerebrospinal fluid cultures revealed *S. suis* serotype 2, which was resistant to tetracycline and clindamycin. Table 1 lists the laboratory data of the patients.

**Table 1:** Laboratory data

Test	Result	Normal range of value
**Blood test results**		
White blood cell count	16.27 × 10^9^/L	(3.5-9.5) × 10^9^/L
Neutrophil ratio	90.8%	(40-75)%
Neutrophil count	14.77 × 10^9^/L	(1.8-6.3) × 10^9^/L
Hemoglobin	141 g/L	(130-175) g/L
Platelet count	128 × 10^9^/L	(125-350) × 10^9^/L
Creatinine	107 µmol/L	(59-104) µmol/L
Serum procalcitonin	32 ng/mL	(0-0.05) ng/mL
Lactic acid	10.2 mmol/L	(0.5-1.7) mmol/L
Glucose	21.3 mmol/L	(3.9-6.1) mmol/L
Cholesterol	5.24 mmol/L	(3.6-5.2) mmol/L
Triglyceride	2.59 mmol/L	(0.45-1.81) mmol/L
Low-density lipoprotein	2.82 mmol/L	(2.7-3.1) mmol/L
AST	22 U/L	(15-40) U/L
ALT	24 U/L	(9-50) U/L
Blood culture	S. Suis serotype 2	-
**CSF analysis**		
Glucose	1.4 mmol/L	(2.5-4.5) mmol/L
Protein	1.46 g/L	(0-0.45) g/L
Chlorid	101 mmol/L	(120-132) mmol/L
White blood cell count	4400 × 10^6^/L	(0-5) × 10^6^/L
Pressure	330 mmH_2_O	(80-180) mmH_2_O
CSF culture	S. Suis serotype 2	-

AST, Aspartateaminotransferase; ALT, Alanine aminotransferase; S. Suis, Streptococcus suis; CSF, Cerebrospinal fluid

**Fig. 1:**
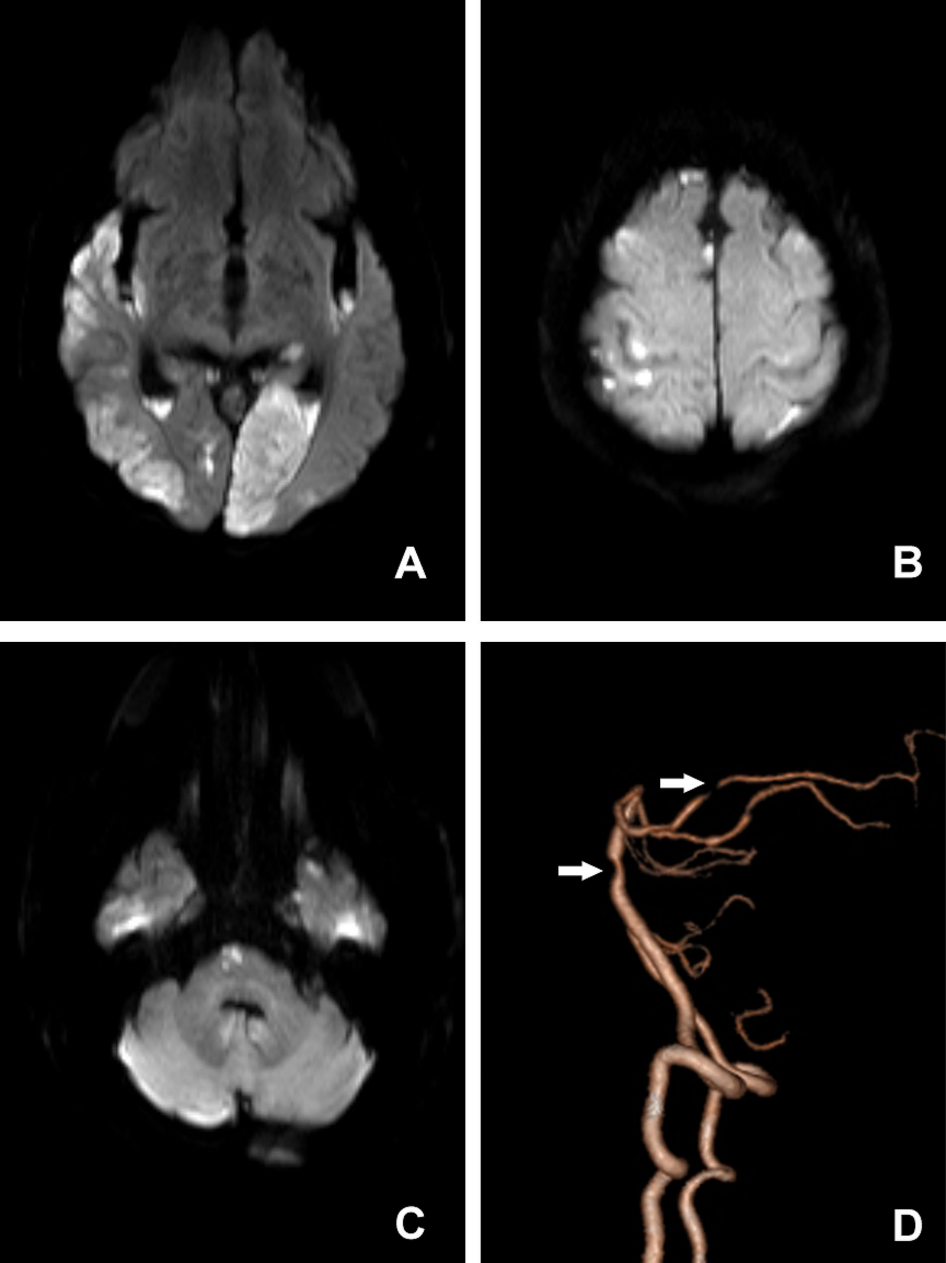
Imaging examination of the patient. Diffusion-weighted magnetic resonance imaging of the brain revealed high signals in the pons and bilateral frontotemporal parietal occipital lobes (**A**, **B**, **C**). Computed tomography angiography showed severe stenosis in the P2 segment of the posterior cerebral artery and moderate stenosis in the basilar artery (**D**)

Brain computed tomography (CT) revealed lacunar cerebral infarction of the pons, left basal ganglia, and bilateral corona radiata. Computer tomography angiography (CTA) revealed severe stenosis in the M2 segment of the left middle cerebral artery and the P2 segment of the posterior cerebral artery; moreover, moderate stenosis was detected in the basilar artery, the A3 segment of the left anterior cerebral artery and the M2 segment of the right middle cerebral artery. The P2 segment of the posterior cerebral artery and the basilar artery are shown in Fig. 1.

At first, the patient was empirically given anti-infection treatment with 2 g of meropenem q8h. To reduce intracranial pressure and inflammation, dexamethasone therapy(20 mg qd) were administered. At the same time, the patient was given antiplatelet treatment with clopidogrel, hypolipidemic treatment with atorvastatin, antihypertensive treatment with amlodipine besylate, sugars were controlled with insulin, and cerebral circulation improvement with butyphthalide. For antishock treatment, blood volume was supplemented and norepinephrine was implemented. A clear diagnosis of *S. suis* meningitis was obtained on 23 September, and the antibiotic treatment was changed to ceftriaxone 3 g q24h combined with penicillin 4.8 million units q6h from 23 September to 26 September. After 5 days of treatment, the patient’s condition was stable, but he was still unconscious. Considering the delayed appearance of cerebral infarction lesions on CT, brain magnetic resonance imaging was performed, which revealed multiple acute cerebral infarctions in the pons and bilateral frontotemporal parietal occipital lobes (Fig. 1). After being weaned off the ventilator and after his state of shock improved, the patient was transferred to the Department of Neurology on 4 October. His temperature decreased to normal from 17 October. Antibiotics were discontinued on 24 October. After treatment, the patient gradually became conscious but showed symptoms of irritability, aggression, and insomnia, and was verbally abusive. He could execute simple commands, such as raising his hands and closing his eyes. He always answered questions wrong and often talked to himself. Olanzapine and alprazolam were initiated to alleviate the symptoms. The patient was transferred to the rehabilitation department on 11 November, and he was discharged on 2 December. At the time of discharge, the patient exhibited cognitive impairment and irritability. The muscle strength of the limbs was grade 4. Due to his cognitive impairment, the patient’s hearing in both ears and Mini-Mental State Examination (MMSE) score could not be checked. His NIHSS score was 4 points, and his modified Rankin scale (mRS) score was 3 points.

## Discussion

*Streptococcus suis*, a heterogeneous Gram-positive bacterium, can cause zoonotic diseases. People who are occupationally exposed to pigs and/or pork are the main risk groups. Contact with pigs or pork products and consuming raw pig meat or blood are important routes of infection. Many reported human cases of *S. suis* infection have been reported worldwide, with approximately 83.6% occurring in Vietnam, Thailand, and China. This has created serious health problems for China [4]. *S. suis* serotype 2 is most closely associated with human infections [3, 5] and may cause a variety of symptoms, including purulent meningitis and sepsis, the most common of which is purulent meningitis. Patients often present with fever, headache, neck stiffness, altered consciousness, and nausea or vomiting [2]. Infections of other a-hemolytic streptococci, such as *Streptococcus pneumoniae*, viridans group streptococci, ‘Group D streptococci’, and enterococci, may cause similar symptoms, and culture, polymerase chain reaction (PCR) and serum agglutination tests can help to differentiate among the various species [1, 3]. The key treatment is the use of antibiotics based on sensitivity testing, and ceftriaxone and penicillin are often chosen [2]. Most patients have a good prognosis, and approximately one-third of patients recover without sequelae [2]. The fatality rate of meningitis caused by S. suis is 2.9% [2].

The most common sequela of patients who have recovered from purulent meningitis caused by *S. suis* infection is hearing loss. Up to one-half of patients indicate hearing loss can occur at presentation or a few days later.

The use of dexamethasone may improve hearing impairment [6]. We attempted to evaluate the patient’s hearing, but more detailed professional tests were not available due to the uncooperative nature of the patient. The presence and extent of hearing loss in our patient was uncertain. In this report, the patient also exhibited cognitive impairment. Compared with hearing loss, cognitive impairment is a rare sequela of *S. suis*, and previous research has suggested that cognitive impairment was present in only 2 of 286 patients (0.70%) [2]. Poststroke dementia is also a concern. However unfortunately, we were unable to assess the extent of the patient’s dementia, and a diagnosis of poststroke dementia could not be made during the current course of the disease. At the time of discharge, the patient still had difficulty communicating and living independently.

In this case, after considering that the patient had meningitis and sepsis, combination therapy with ceftriaxone and penicillin was given. Streptococcus suis is generally susceptible to a variety of antibiotics, such as penicillin, ampicillin, amoxicillin, flucloxacillin, cephalosporin and ceftriaxone [3, 7]. Ceftriaxone and penicillin are most often selected for treatment [2]. However, previous studies have suggested that the use of penicillin and ceftriaxone alone is not always effective for severe cases [8, 9]. For severe cases, combination therapy with multiple antibiotics is reasonable [1].

Ischemic stroke is a common complication of bacterial meningitis and occurs in 10–29% of cases [10]. However, *S. suis* complicated with acute cerebral infarction has rarely been reported. Xing et al. [11] reported a case of *S. suis* meningitis with acute cerebral infarction. However, it was a lacunar cerebral infarction, and after treatment, the patient had no obvious neurological sequelae except for hearing loss in both ears. Previous research has suggested that *Streptococcus* causes cerebral ischemia by augmenting atherosclerosis, causing systemic inflammation [12], and promoting thrombosis [13]. Autopsies of patients who died of *S. suis* infection revealed multiple organ hemorrhage, cerebral edema, leptomeningeal congestion and disseminated intravascular coagulation [14]. These changes may promote cerebral ischemia. It is worth noting that the patient suffered from hypertension, diabetes and hyperlipidemia, and the CTA also showed that his cerebral blood vessels had multiple stenosic lesions. This patient was at a high risk of cerebral infarction, and we hypothesize that *S. suis* infection may be a trigger for cerebral infarction rather than a major cause; however more research is needed in the future to determine this relationship.

## Conclusion

In the present study, we reported a case of cerebral infarction associated with *S. suis* infection. For anti-infection treatment, meropenem, penicillin, and ceftriaxone were used and the treatment lasted from September 22 to October 24. After treatment, the patient exhibited cognitive impairment, dyspraxia and irritability. There are limited case reports of cerebral infarction associated with *S. suis* infection, and further research is needed to determine the best treatment method.

## Data Availability

Data are available on request from the corresponding author upon reasonable request.
